# Evaluation of a community pharmacy-based intervention for improving patient adherence to antihypertensives: a randomised controlled trial

**DOI:** 10.1186/1472-6963-10-34

**Published:** 2010-02-05

**Authors:** Rosalind Lau, Kay Stewart, Kevin P McNamara, Shane L Jackson, Jeffery D Hughes, Gregory M Peterson, Diana A Bortoletto, Jenny McDowell, Michael J Bailey, Arthur Hsueh, Johnson George

**Affiliations:** 1 Centre for Medicine Use and Safety, Department of Pharmacy Practice, Faculty of Pharmacy and Pharmaceutical Sciences, Monash University, 381 Royal Parade, Parkville, VIC 3052, Australia; 2 Greater Green Triangle University Department of Rural Health, Flinders and Deakin Universities, Deakin University, Princes Hwy, Warrnambool, VIC 3280, Australia; 3 Unit for Medication Outcomes Research and Education, School of Pharmacy, University of Tasmania, Churchill Avenue, Sandy Bay, TAS 7005, Australia; 4 School of Pharmacy, Curtin University, Kent Street, Bentley, WA 6102 Perth, WA 6845, Australia; 5 Department of Epidemiology and Preventive Medicine, Monash University, Alfred Hospital, Commercial Road, VIC 3004, Australia; 6 Centre for Health Policy, Programs and Economics, School of Population Health, The University of Melbourne, 207 Bouverie Street, Carlton, VIC 3053, Australia

## Abstract

**Background:**

The majority of patients using antihypertensive medications fail to achieve their recommended target blood pressure. Poor daily adherence with medication regimens and a lack of persistence with medication use are two of the major reasons for failure to reach target blood pressure. There is no single intervention to improve adherence with antihypertensives that is consistently effective. Community pharmacists are in an ideal position to promote adherence to chronic medications. This study aims to test a specific intervention package that could be integrated into the community pharmacy workflow to enable pharmacists to improve patient adherence and/or persistence with antihypertensive medications - Hypertension Adherence Program in Pharmacy (HAPPY).

**Methods/Design:**

The HAPPY trial is a multi-centre prospective randomised controlled trial. Fifty-six pharmacies have been recruited from three Australian states. To identify potential patients, a software application (MedeMine CVD) extracted data from a community pharmacy dispensing software system (FRED Dispense^®^). The pharmacies have been randomised to either 'Pharmacist Care Group' (PCG) or 'Usual Care Group' (UCG). To check for 'Hawthorne effect' in the UCG, a third group of patients 'Hidden Control Group' (HCG) will be identified in the UCG pharmacies, which will be made known to the pharmacists at the end of six months. Each study group requires 182 patients. Data will be collected at baseline, three and six months in the PCG and at baseline and six months in the UCG. Changes in patient adherence and persistence at the end of six months will be measured using the self-reported Morisky score, the Tool for Adherence Behaviour Screening and medication refill data.

**Discussion:**

To our knowledge, this is the first research testing a comprehensive package of evidence-based interventions that could be integrated into the community pharmacy workflow to enable pharmacists to improve patient adherence and/or persistence with antihypertensive medications. The unique features of the HAPPY trial include the use of MedeMine CVD to identify patients who could potentially benefit from the service, control for the 'Hawthorne effect' in the UCG and the offer of the intervention package at the end of six months to patients in the UCG, a strategy that is expected to improve retention.

**Trial Registration:**

Australian New Zealand Clinical Trial Registry ACTRN12609000705280

## Background

A continuous positive correlation exists between increasing blood pressure (BP) and mortality rates [1]. The cardiovascular mortality benefit associated with achieving BP control through medications has been demonstrated through large-scale clinical trials [2,3]. Unfortunately, the majority of patients on antihypertensive medications fail to achieve their recommended target BP [4-6]. Poor adherence with medication regimens and a lack of persistence with medication use are two of the major reasons for failure to reach target BP [6]. Several antihypertensive medication trials have found persistence after one year to be less than 50% [7-9]. On any day, patients who were still engaged with the drug dosing regimen omitted about 10% of the scheduled doses, of which 42% were of a single day's dose and 43% were part of a sequence of several days [7]. Adherence and persistence are likely to be much worse in routine clinical practice where, unlike clinical trials, patients' use of medications is not closely monitored.

There is no single intervention to improve adherence with antihypertensives that is consistently effective. Medication nonadherence is a multi-factorial issue. Interventions, either individually or in combination, for improving adherence with antihypertensives that have some evidence base are: simplifying dosing regimens, motivational strategies, unit dose packaging, educational counselling over the telephone, refill reminders, self-monitoring, and dose-tailoring [10-13]. As medicines experts in primary care, community pharmacists are in an ideal position to address adherence and persistence issues in people with hypertension. In a US study of 200 community-dwelling elderly patients, a 36% absolute increase in medication adherence followed a comprehensive pharmacist intervention with educational and structural components, which was also associated with improved cardiovascular outcomes [14]. The educational component included intensive and frequent counselling by a pharmacist, while the structural component involved packaging of medications in blister packs that contained each patient's daily medications. Australian community pharmacists are uniquely positioned in the healthcare system to undertake a role in cardiovascular medication management, a role supported by public opinion [15]. However, the pharmacist's role in addressing intentional and unintentional nonadherence to antihypertensive medicines in a sample of patients at risk of nonadherence remains untested using a randomised controlled design.

### Aim of the research

To test a specific intervention package that could be integrated into community pharmacy workflow to enable pharmacists to improve patient adherence and/or persistence with antihypertensive medications - Hypertension Adherence Program in Pharmacy (HAPPY).

### Primary outcome measures

The primary outcomes of the HAPPY trial are changes in patient adherence and persistence at the end of six months. These will be measured subjectively using the self-reported Morisky scale [16] and the Tool for Adherence Behaviour Screening (TABS) [17] and objectively using the medication refill data (e.g. MedsIndex score [18]). The Morisky scale assesses both intentional and unintentional nonadherence and comprises four items. Responses for each item are scored 0 for 'yes' and 1 for 'no' (except for the item 'are you always careful in taking medicines?' where the score was reversed) and added together. A total score of zero represents good adherence and a score of one or more represents suboptimal adherence. The TABS is another self-reported adherence measure, which was originally developed in Australian patients using chronic medicines. It has two subscales - 'adherence' and 'nonadherence' - each comprising four items to be answered on a 5-point Likert-type scale ('never' - 1 to 'always' - 5). The total score for 'nonadherence' will be subtracted from that of 'adherence'; a differential of ≥15 will be regarded as good adherence and ≤14 will be regarded as suboptimal adherence. The TABS measures both intentional and unintentional deviations from recommended management and has been shown to have greater incremental validity than other self-reported adherence measures [17]. The MedsIndex gives each patient a score out of 100 for each of their long-term medications, which is calculated based on their refill history. Any score less than 100 suggests suboptimal adherence.

### Secondary outcome measures

Secondary outcomes include changes in patients' BP control, satisfaction with and willingness to pay for the service and economic benefits.

## Methods/Design

### Study Design

The HAPPY trial is a multi-centre prospective randomised controlled trial (RCT).

### Study Setting

Community pharmacies included in the trial come from metropolitan, regional and remote areas in three states of Australia (Victoria, Western Australia and Tasmania), proportionally stratified according to Pharmacy Access/Remoteness Index of Australia (PhARIA) [19] categories. (PhARIA provides a comprehensive, standardised measurement of the physical and professional remoteness of pharmacies throughout Australia, for use in the determination of rural and remote pharmacy allowances. The concept of remoteness was based on the road distance people have to travel to reach a range of services.) The pharmacies are located in a range of physical locations such as shopping strips, shopping malls, within medical centres and stand alone.

### Pharmacy Eligibility Criteria

Pharmacies that met all the following criteria were eligible to participate in the HAPPY trial:

• use the pharmacy dispensing program 'FRED Dispense^®^';

• agree to install a software application (MedeMine CVD) to identify patients who are using or have used antihypertensive medicines in the previous six months;

• have at least 40 patients who are currently using or have used antihypertensive medicines in the previous six months;

• have a private counselling area within the pharmacy where one-on-one consultation with a patient will be shielded from the public eye and ear;

• willingness and ability to attend project training either face-to-face or online;

• have time within the working week to allocate about one uninterrupted hour per patient to collect the baseline data; and

• able to follow-up participants for at least six months from baseline.

### Recruitment of pharmacies

Contact information for pharmacies was obtained from the telephone directory/Internet. Pharmacies in each of the PhARIA categories in each state were contacted by telephone and informed about the project. An explanatory statement and consent form was sent to all eligible pharmacies, if the pharmacist expressed interest. Upon receipt of the signed consent form, pharmacies were formally included in the study.

### Cluster randomisation

In each state, pharmacies were randomised to either Pharmacist Care Group (PCG) or Usual Care Group (UCG) according to the PhARIA. Randomisation was carried out at the pharmacy level to avoid the contamination likely to result from the same pharmacy recruiting and following up both intervention and control group patients. Randomisation was carried out at a central location using the sealed opaque envelope technique. To check for any 'Hawthorne effect' [20,21] in the UCG due to the effect of data collection, a third group of patients (Hidden Control Group - HCG) who are taking or have taken antihypertensives in the previous six months (but not included as participants in the UCG) will also be identified.

### Pharmacist Care Group (PCG)

The PCG participants receive a package of interventions from the pharmacist for enhancing their antihypertensive medication adherence, which includes:

• a home BP monitor (Omron^®^T9IT) with the capacity to store and download BP readings to be used for discussion at three- and six-month follow-ups;

• training by the pharmacist on self-monitoring of BP;

• motivational interviewing and education by the pharmacist to help patients improve their medication adherence and achieve target BP;

• pharmacist-initiated home medicines review (HMR), dose administration aid (DAA) and/or patient medication profile (PMP), where necessary;

• medication use review (MUR) to identify and resolve possible medication-related hypertension (e.g. due to non-steroidal anti-inflammatory drugs, cold preparations, complementary medicines, etc.);

• referral to a GP when needed (e.g. very high blood pressure); and

• refill reminders (by either SMS, telephone or mail) from their pharmacist at a chosen number of days before their antihypertensive medication dispensing is due.

HMRs http://www.medicareaustralia.gov.au/provider/pbs/fourth-agreement/hmr.jsp are designed to assist consumers living at home to maximise the benefits of their medication regimen and prevent medication-related problems. The review involves the consumer's general practitioner (GP) and preferred community pharmacy, and in some cases other relevant members of the healthcare team. The GP refers the consumer to the community pharmacy and an accredited pharmacist visits the consumer at home, reviews their medication regimen, and provides the GP with a report. The GP and consumer then agree on a medication management plan.

A DAA http://www.health.gov.au/internet/main/publishing.nsf/Content/ppsac-qa-daa is a device developed to assist patients in better managing their medicines by arranging their medicines into individual doses according to the prescribed dose schedule throughout the day. The aim of the DAA Program is to reduce medication-related hospitalisation and adverse events through improving medication management and adherence for people in the community.

A PMP http://www.health.gov.au/internet/main/publishing.nsf/Content/ppsac-qa-pmp is a comprehensive written summary of all regular medicines taken by a patient that assists them in understanding and managing their medicines by informing them how, when and why to take their medicines. The aim of the PMP Program is to reduce the risk of medication-related adverse events by assisting people to understand and manage their medications, including prescription, over-the-counter and complementary medicines.

A MUR http://www.psnc.org.uk/pages/murs_the_basics.html usually takes place in the pharmacy and it involves the pharmacist checking the patient's medication, making sure that the patient knows how and why they should be taking their medication, as well as identifying any problems. It provides the patient with an opportunity to ask questions and the pharmacist an opportunity to improve the patient's medication understanding and adherence, as well as being able to highlight problems and provide appropriate solutions.

### Usual Care Group (UCG)

UCG pharmacists will continue to offer routine care to their patients.

### Training for Pharmacists

Training was organised for all participating pharmacists in two modes, face-to-face and online. Materials and resources were made available as hard copy, online and on CDs, to provide flexible learning opportunities. Training comprised two phases:

### Phase I

Phase I training was delivered to all participating pharmacists and included the project overview, patient recruitment including the use of MedeMine CVD software to identify patients using antihypertensive medications, calculation of MedsIndex score, use of BP monitors and collection of baseline data.

### Phase II

The second phase focused on the importance of medication adherence in achieving optimal BP control, a clinical update on hypertension management, motivational interviewing, review of BP monitoring by patients, downloading home BP recordings, charting BP against adherence, and identification and resolution of medication-related hypertension by MUR.

Pharmacists in the PCG were offered Phase II training before baseline data collection. The UCG pharmacists will receive Phase II training after the six-month follow-up, so that control patients can be offered the interventions tested in the PCG after the study. This will encourage recruitment of pharmacists to the UCG and improve retention of patients in the UCG.

### Sample size

Australian, European and North American studies have estimated that around 50% of patients initiating an antihypertensive medication discontinue their medication (become nonpersistent) within twelve months [22]. To demonstrate a 15% difference in adherence between the control and intervention groups at six months (e.g. 50% in the UCG versus 65% in the PCG) with 80% power and a two-sided p-value of 0.05, 182 patients are required per study group. To allow for potential drop-outs (approximately 25% over six months) 225 patients need to be recruited per group; i.e. a total of 450 patients at baseline.

### Recruitment of patient participants

A software application, MedeMine CVD, has been installed on the dispensing computer of participating pharmacies. MedeMine CVD extracts data from the market leading pharmacy dispensing software system in Australia (FRED Dispense^®^) and identifies patients who are using or have used antihypertensive medicines in the previous six months. The purpose of MedeMine CVD is to:

1. preferentially identify patients who last had their antihypertensive medications dispensed more than 59 days previously and those with suboptimal refill adherence;

2. generate personalised "Expression of Interest" (EOI) forms to mail to potential patient participants. Once the completed EOI has been received by the pharmacist and eligibility is confirmed, the information is recorded in MedeMine CVD;

3. generate the participant explanatory statement and consent form, as well as a letter and fax-back form to be sent to the participant's GP to confirm diagnosis of primary hypertension and advise of the participant's target BP;

4. manage participant appointments by pharmacists;

5. create a unique identification number for each participant, which is printed on all study documents (e.g. data collection forms and letters); and

6. send SMS reminders or prompt the pharmacist to telephone or send mail reminders, to participants who have requested this part of the service, when their supplies are dwindling.

An invitation letter (EOI) with a short questionnaire (seeking information about the patient's BP, interest in participation, nominated GP, including contact details and consent to contact the GP, and patient telephone contact details) and a reply-paid envelope were sent to potential participants. Patients were asked to return the completed questionnaire to the pharmacy and indicate their interest in participating. Once the EOI is received, the pharmacist will contact the patient to further assess their eligibility and arrange a mutually convenient time to visit the pharmacy to enrol in the study. If eligibility is confirmed, the consent form would then be signed. The pharmacist will also contact the patient's GP. At no point are patients' identities made known to the researchers.

### Inclusion criteria

• using or having used in the last six months at least one antihypertensive medication belonging to the four most common classes of antihypertensives in Australia - angiotensin converting enzyme inhibitors angiotensin-II receptor antagonists, calcium channel blockers, beta blockers -  http://www.aihw.gov.au/publications/cvd/mfchatua/mfchatua.pdf or fixed combinations of antihypertensive medications belonging to the above classes with other antihypertensives (e.g. diuretics);

• a diagnosis of primary hypertension;

• 18 years and above; and

• available for follow-up for at least six months from baseline.

### Exclusion criteria

• participation in other adherence promotion programs;

• having had a pharmacist-conducted HMR service in the last 12 months;

• unable to communicate in English; and

• does not self-administer antihypertensive medicines.

Each pharmacist was asked to recruit 7-10 patients from their pharmacy.

### Data collection

### Baseline visit

During the baseline visit to the pharmacy, the pharmacist will measure the patient's BP using a digital BP monitor (average of two readings as per WHO MONICA [23] and EHRM [24] protocols - if these differ by more than 5 mmHg, a third reading will be taken and the average of the two closest readings will be recorded). Each pharmacy has been provided with a BP monitor. The MedsIndex score will be calculated for each antihypertensive medicine the patient uses. Adherence will be measured using self-report tools - the Morisky scale and the TABS. The Beliefs and Behaviour Questionnaire [17] will be used to assess patient experiences, attitudes and beliefs regarding their health and treatment and the 8-item short Assessment of Quality of Life (AQoL) [25] will be used to measure health-related quality of life. The pharmacist will also collect information on the patient's medications and their understanding of and ability to use these medications. The PCG will also receive the intervention described earlier.

### Three-month visit

#### Pharmacist Care Group (PCG) Only

During the patient's visit to the pharmacy at three months, the pharmacist will administer the Morisky scale and TABS. The pharmacist will also:

• download the patient's home BP measurements for review and discussion with the patient;

• measure patient's BP (as described above);

• document any adherence issues and actions taken; and

• refer patient to their GP, if needed (e.g. persistent uncontrolled BP even after improvement in adherence).

### Six-month visit (Final project visit)

Measures performed at baseline will be repeated for both UCG and PCG patients at their six-month visit to the pharmacy.

#### Usual Care Group (UCG) Only

Following final measurements, each UCG participant will receive the same package of interventions offered to the PCG at baseline. UCG participants will receive follow-up pharmacy services two months after the 'Final project visit'. The pharmacist will:

• measure patient's BP (as described above);

• review patient's home BP recordings;

• review patient's BP measurement techniques;

• discuss adherence issues; and

• refer when needed (e.g. persistent uncontrolled BP).

### Data analysis

Participant characteristics in the UCG and PCG will be compared at baseline. Paired t-tests and/or Wilcoxon Signed Rank test will be used to compare changes in total scores on the Morisky scale, TABS and MedsIndex between the PCG and the UCG at baseline and six months. MedsIndex scores will be compared using paired t-tests and/or Wilcoxon Signed Rank test in the UCG and the HCG at six months.

Baseline data will be used to identify factors associated with suboptimal adherence in the whole study population. Significant factors in univariate analysis (chi-square, Mann-Whitney U test and Student's t-test) will be further tested in multivariate analysis (linear regression) to identify independent predictors of nonadherence/nonpersistence in the study population. To adjust for cluster randomisation, a pharmacy variable will also be included in the multivariate models.

An economic evaluation will also be carried out and will be reported elsewhere.

### Ethics

This project has been approved by the Human Research Ethics Committees of Monash University, Curtin University and The University of Melbourne, and the Human Research Ethics Network of Tasmania. All pharmacists and patient participants (in the UCG and PCG) provided written informed consent at the time of enrolment.

Approval to access dispensing data for the HCG was given on the basis that pharmacists have routine access to this data about their patients via the dispensing program and would use it in normal practice to routinely check their patient's history for actual or potential medication-related problems. The researchers will have access only to deidentified patient data.

### Study timeline

Trial start: June 2009

Baseline data collection: June-August 2009

Three-month data collection: October-November 2009

Six-month data collection: December 2009-January 2010

Data analysis: February 2010

## Discussion

To our knowledge, HAPPY trial is the first RCT testing a comprehensive package of evidence-based interventions able to be integrated into the community pharmacy workflow, to enable pharmacists to improve patient adherence and/or persistence with antihypertensive medications. HAPPY trial is unique because we have taken into consideration the 'Hawthorne effect' in the UCG by having a third group of patients (HCG) who are taking or have taken antihypertensives in the previous six months but not included in the UCG. In addition, UCG pharmacists and their patient participants will not be disadvantaged as, after the study period is completed, they will be offered the intervention package tested in the PCG, a strategy that is expected to enhance participant retention.

## Trial registration

Australian New Zealand Clinical Trial Registry ACTRN12609000705280

## Abbreviations

BP: Blood pressure; TABS: Tool for Adherence Behaviour Screening; RCT: Randomised Controlled Trial; PhARIA: Pharmacy Access/Remoteness Index of Australia; CVD: Cardiovascular disease; PCG: Pharmacist Care Group; UCG: Usual Care Group; HCG: Hidden Control Group; HMR: Home medicines review; DAA: Dose administration aid; PMP: Patient medication profile; and MUR: Medication use review; GP: General practitioner; EOI: Expression of Interest; MONICA: Monitoring of trends and determinants in cardiovascular disease; EHRM: European health risk monitoring; and AQoL: Assessment of Quality of Life.

## Competing interests

The authors declare that they have no competing interests.

## Authors' contributions

RL is the project manager of Happy Trial and wrote the first draft of the manuscript with input from JMcD, DB and JG. KS, KMcN, SJ, JH, GP, JMcD, MB, AH and JG were involved in the conception and design of the study and also have an ongoing involvement in the management of the study. All the authors read and approved the final manuscript.

## Pre-publication history

The pre-publication history for this paper can be accessed here:

http://www.biomedcentral.com/1472-6963/10/34/prepub
